# How to apply case reports in clinical practice using surrogate models via example of the trigeminocardiac reflex

**DOI:** 10.1186/s13256-016-0849-z

**Published:** 2016-04-06

**Authors:** Nora Sandu, Tumul Chowdhury, Bernhard J. Schaller

**Affiliations:** 1 Departement of Research, University of Southampton, University Road, Southampton, SO17 1BJ UK; 2 Department of Anesthesiology and Perioperative Medicine, University of Manitoba, 671 William Avenue, Winnipeg, MB R3E 0Z2 Canada

**Keywords:** Trigeminocardiac reflex, Skull base surgery, Case report, Model thinking

## Abstract

Case reports are an increasing source of evidence in clinical medicine. Until a few years ago, such case reports were emerged into systematic reviews and nowadays they are often fitted to the development of clinical (thinking) models. We describe this modern progress of knowledge creation by the example of the trigeminocardiac reflex that was first described in 1999 by a case series and was developed over the cause-and-effect relationship, triangulation to systematic reviews and finally to thinking models. Therefore, this editorial not only underlines the increasing and outstanding importance of (unique) case reports in current science, but also in current clinical decision-making and therefore also that of specific journals like the *Journal of Medical Case Reports*.

## Introduction

Learning is a continuum process of knowledge creating. In the scientific world, such knowledge can be acquired and understood by various sources available in the scientific literature including meta-analysis, systematic reviews, randomized control trials, clinical studies, case reports, editorials, letters, and expert comments. However, case reports are generally meant to describe the one, often very specific, aspect of a scientific problem or solution, and thus were considered to impose limited utility on common day-to-day practice in the past. However, during the last few years, numerous clinical case reports have been published at a high scientific level so that a new genre has been established and many scientific journals are mainly focused and titled based on these reports [1]. Thus, these case reports are now a generally accepted method of scientific communication of new, challenging and rarer clinical findings to the medical scientific community [1, 2]. Furthermore, case reports can raise useful questions about theories in conclusion of a single clinical observation, and provide, therefore, preliminary data to be tested in investigations that are more rigorous. But there is still a need to create clinically usable knowledge from such case reports, as they are generally “only” the report of a very specific, rare and therefore often not wholly reproducible case [3]. Loftus and Guyer [4] conclude that case reports “illuminate, but can also obscure the truth”. In the majority of case studies, the major goal is to generate and test the hypothesis, “not as answers to questions” [4]. On the other hand, despite its low reliability of evidence for clinical decision-making, there are many instances where valuable knowledge has come from someone taking the trouble to write up cases that are out of the ordinary [5–10]. When such case reports are published, they may alter other physicians’ decision-making and may stimulate further investigations that are more generalizable. One such key example is represented by the trigeminocardiac reflex (TCR). The TCR, a well-recognized brainstem reflex consisting of bradycardia, arterial hypotension, apnea and gastric hypermobility [5], was first seen in one neurosurgical case, then published, for the first time, as a small case series in 1999 [5] and was followed by various small case series or case reports over the last nearly 20 years [11–18]. Clear and comprehensive proof of this reflex’s existence was provided on a cause-and-effect relationship basis that is always based on a single case [1, 5]. After this initial case series that also suggested, for the first time, a definition of the TCR based on clinical and theoretical consideration, the subsequently published case reports could therefore focus, related always to this first proof of existence, on the differentiation between the peripheral [17–20, 21, 22], central [7, 19] or ganglion stimulation [18, 23, 21]. Like so, only based on case reports, a differentiation to stimulation by peripheral-central-ganglion Gasseri brainstem stimuli could be ascertained, [18, 21–25]. Therefore, TCR can be considered not only as an ideal example of the utility of the case report but also of development of a clinical topic and therefore knowledge creation in the scientific literature based solely on case reports.

### Triangulation as a solution

There is another, in the last year increasing, circumstance in which authors should write, and journals should publish descriptive case reports or case series: this is in conditions where population-based studies or treatment trials are difficult or impossible to organize. In the current overregulated research community, it is more and more difficult and expensive to perform prospective studies or trials. However, in such circumstances small case series are a solution out of this increasing dilemma, but they often have the problem of generalizing the newly reported and created knowledge. Nevertheless, the qualitative method of “triangulation” [26, 27] through cross-verification helps in such circumstances.

Therefore, a critical reflexivity is absolutely essential in case reports/series, which was resolved in the example of the TCR by performing a causal relationship test in the case of the initial TCR publication [1, 5, 20, 28, 29]. Thus, TCR can be prevented by avoiding stimulation of the afferent pathway or by blocking the nerves that conduct the afferent impulses [30–35]. Such a cause-and-effect relationship, on the one side, is evidence of a TCR, and the stimulation of the trigeminal root, on the other side, represents as well as confirms the afferent pathway [36, 37]. The few single-case reports that followed the years after the initial TCR report in 1999 were therefore also important to rigorously test our first established hypothesis of the existence of this newly described reflex [38–45]; all done by the method of triangulation. If just one such observational case does not fit completely with the initial proposition, the hypothesis should be considered generally not valid and therefore should be revised or even rejected [1, 13]. This was not the case with our hypothesis of the existence of the TCR [13, 17, 35, 38] and demonstrates that – besides the qualitative method of triangulation – the ongoing description of similar cases about a specific topic not only has importance for clinical practice but also for the scientific community.

### Limitations of descriptive studies for knowledge generalization

On the other hand, it must be admitted that small or single descriptive studies have serious limitations [1]. One is that retrospective description of case reports is rarely complete [1]. Who can say whether the outcomes of the missing cases might have been very different leading to another conclusion? [1]. Another is that quirks in the way that unusual cases are referred make it hard to feel confident in generalizing from the experience of one center or one single author in a very limited time span [1]. A third, Grimes and Schulz have pointed out [46] that without a comparison group, which is seldom the case in case report/series, causal inferences about temporal associations need to be treated with deep suspicion. However, case reports are notwithstanding especially suited for such further scientific investigations and theory building in clinical medicine because of their in-depth approach based on the specific finding of a special case [1]. For this reason, it was additionally tested whether skull base operations, other than for vestibular schwannoma, also demonstrated an intraoperative occurrence of the TCR, and we finally found that the phenomenon could also be seen during transsphenoidal surgery [8, 12, 15, 38], during Janetta operations [11], during aneurysm clipping [14], during evacuation of chronic subdural hematoma [1, 41], or other skull base procedures [31]. The TCR is therefore not a seldom but a ubiquitous phenomenon in skull base and facial surgery. Because this issue of generalization has appeared in the (scientific) medical literature with regularity in the last nearly 20 years, we have conducted further TCR-orientated research in different skull base approaches to facilitate and also underline this generalization (see for example [47, 48]). Further case reports and case series have confirmed the ubiquitous phenomenon and have underlined its importance as an autonomic reflex [17], but they have also pointed out the functional importance of the TCR on the surgical outcome after skull base surgery. Focusing on hearing function in patients with vestibular schwannoma has demonstrated that the hypotension owing to the TCR to be a prognostic factor for hearing preservation, postoperative tinnitus or anatomical location [17, 24, 31]. Such facts gained from the experience of the TCR research point out that extreme or atypical cases often reveal more detailed information because more basic mechanisms of the disease are activated in these specific circumstances than is the case in larger and often multicenter trials [22, 30]. In addition, from both an understanding-oriented and an action-oriented perspective, it is often more important to clarify the deeper cause behind a given clinical picture and its consequences than to describe only signs and symptoms of a disease and how frequently it occurs as we see it often in larger trials [1, 16], even though there remains a lack of generalization.

### Systematic reviews

Systematic reviews of observational studies are much harder to carry out and interpret than systematic reviews of randomized controlled trials. The main difficulties lie in coping with the wide diversity of study designs used by different investigators, and the biases inherent in most observational studies. Therefore, better and more appropriate scientific methods are still being developed and argued over [1], but it is already clear that applying an evidence-based approach to traditional descriptive studies is more and more useful in a clinical-orientated context in the new millennium. For example, a few case reports on the TCR, published after 2000, inform about some risk factors for the intraoperative occurrence of the TCR [1, 17, 20, 28, 34, 35]. Several factors have been postulated as predisposing patients to the TCR based on a few such clinical case reports [20, 21, 35]. These factors include hypercapnia, hypoxemia, light anesthesia, high resting vagal tone in children, narcotics such a sufentanil and alfentanil, preoperative beta blockers and calcium channel blockers [20]; most of these “risk” factors are rather anecdotal than really scientifically proven. Therefore, cases such as the above-cited critical “follow-up cases” can have more importance in relation to the general clinical feature of the initial hypothesis than to merely represent a small sample of patients. If such “follow-up cases” are lacking, the single report of a case retains some anecdotal character and must be considered of only questionable clinical use. However, if the initial hypothesis also applies in these “follow-up cases,” it could also be expected that the initial hypothesis is to be applicable to a large range of patients. In this manner, by strategically selecting cases, one can arrive at the point at which cases allow generalization as a particular research methodology [48, 49]. Also, with more uniform considerations in reporting the TCR cases, case series and clinical trials, such statistical analyses of the case report literature would become easier and faster [35] with a more and more sound scientific background. Recently, such a systematic review – for the first time for the TCR – was done for different anesthetic techniques in case of TCR occurrence, making the confusing existing literature clearer and making some specific treatment recommendations [35].

### Development of (thinking) models

Nevertheless, in a more complex world and in times of personalized medicine [2], systematic researches alone are often of limited value for daily use in a clinical context. Therefore, development of clinical models is needed. Of course, models are a simplification of the reality and therefore often several models are used to describe a certain medical picture. Recently, such a clinical thinking model was published for the definition of the TCR [21] and later of the pooled prevalence related to neuroanatomical structures [50]. This was an important step in the evolution of TCR research, as evidence shows that people who think with models consistently outperform those who do not. And moreover, people who think with lots of models outperform people who use only one. Why do such clinical models, as recently developed for the TCR [51], make us better thinkers? Models help us to better organize information – to make sense of that fire hose or hairball of data (choose your metaphor) available on the Internet. Models improve our abilities to make accurate forecasts. They help us to make better decisions and to adopt more effective clinical strategies. Models are therefore nowadays what systematic reviews were 10 years ago.

Case reports are especially suited for clinical model development as they often go deeper into clinical details and allow, therefore, the statistical fit of the model with the reality. Especially in the case of TCR, with predominantly case reports that created the current knowledge about the TCR (see for example [50, 52, 53]), the development of clinical models might be the next step in knowledge development about the reflex for the near future.

### TCR and case reports: a success story!

The TCR is now accepted all over the world based on our initial definition in 1999 and is considered one of the well-established complications of modern skull base and most recently spinal surgery (see, for example, [20]). This unique example depicts the outstanding importance of case reports to improve medical knowledge in the scientific (medical) literature. We hope that this success story may inspire others to publish and especially to thoroughly analyze their special cases that deal with not-yet-published clinical features and pictures; this is one of the most important ways that medicine can advance. In addition, our experience and reports underline the importance of special journals such as the *Journal of Medical Case Reports*, which is the leading journal in the field and deals mainly with case reports, and of making great efforts to achieve a high quality of publication in case reports and to stimulate authors to analyze their special cases. The story of the TCR has shown and will continue to show new pathways to explore the usefulness of case reports in the scientific world.

## Conclusions

Whether or not case reports would impart substantial knowledge in medical literature is debatable. However, knowledge of rarer, clinically important reports does influence overall clinical management or decision-making that may affect morbidity and mortality. In this context, TCR case reports have substantially influenced understanding of skull base surgeries, and other neurosurgical as well as neurological diseases. Hence, it gives us a new thinking model for applying knowledge of case reports to the complexity and personalized care needed in daily clinical practice.
